# Humans judge faces in incomplete photographs as physically more attractive

**DOI:** 10.1038/s41598-019-56437-4

**Published:** 2020-01-10

**Authors:** Diana Orghian, César A. Hidalgo

**Affiliations:** 1 CICPSI, Faculdade de Psicologia, Universidade de Lisboa, Alameda da Universidade, Lisboa, 1649-013 Portugal; 2 ANITI Chair, University of Toulouse, 41 Allée Jules Guesde, Toulouse, 31000 France; 3 Alliance Business School, University of Manchester, Booth St W, Manchester, M15 6PB United Kingdom; 4 School of Engineering and Applied Sciences, Harvard University, 29 Oxford St, Cambridge, MA 02138 USA; 5 Datawheel, 1299 Cambridge Street, Cambridge, MA 02139 USA

**Keywords:** Social behaviour, Human behaviour

## Abstract

Attractive people are perceived to be healthier, wealthier, and more sociable. Yet, people often judge the attractiveness of others based on incomplete and inaccurate facial information. Here, we test the hypothesis that people fill in the missing information with positive inferences when judging others’ facial beauty. To test this hypothesis, we conducted seven experiments where participants judged the attractiveness of human faces in complete and incomplete photographs. Our data shows that—relative to complete photographs—participants judge faces in incomplete photographs as physically more attractive. This positivity bias is replicated for different types of incompleteness; is mostly specific to aesthetic judgments; is stronger for male participants; is specific to human faces when compared to pets, flowers, and landscapes; seems to involve a holistic processing; and is stronger for atypical faces. These findings contribute to our understanding of how people perceive and make inferences about others’ beauty.

## Introduction

Often, people judge the appearance of others using incomplete information. Such is the case when we see someone for the first time from far away or in poor light conditions. Similarly, most online encounters involve aesthetic judgements based on small, incomplete, or partly occluded profile pictures^1^. These online personas are important. For example organizations are increasingly using social media (e.g., Facebook, LinkedIn) to gather information about job candidates and the inherent incompleteness of online images can therefore bias the first impressions of employers about potential employees^2^. Here, we conducted a series of experiments to explore how people infer facial attractiveness from incomplete, small, and blurry pictures. The results suggest that, under information shortage, people are positively biased when judging others’ facial attractiveness. This suggests that people fill in the missing information with optimistic inferences.

Positive biased are common in the human cognition. People are known to perceive themselves in unrealistically positive ways^3^. People believe they have more control over the environment than they in fact do^4^, expect a better future than the one predicted from base-rates^4^, and overestimate the prevalence of their own opinions^5^. When comparing themselves with others, the so called “better-than-average” effect suggests that people perceive themselves as kinder, warmer, and sincerer than the average person^6^. These positive illusions, or biases, have a self-serving role: promoting psychological well-being by creating a positive self-image. Yet, all of these effects describe biases that inflate people’s self-perception. Could similar biases also affect people’s perception of others?

There are good reasons to believe that people may have unrealistic expectations when perceiving others as well. The literature has shown that people tend to have optimistic impression about others’ personalities when they have limited information about them^7–9^. Moreover, the excitement of anticipating a first encounter can further amplify these positive expectations^10,11^. But do these biases also apply to physical appearance? Do we perceive others as better-looking when we are presented with incomplete information about their faces?

Such a positivity bias can have profound implications on our social interactions. People that we perceive as more attractive are also perceived as more sociable^12^, healthier and wealthier^13^, academically brighter^14^, and as having more expertise^15^ and better job qualifications^16^. This halo-effect plays an important role not only on how we perceive others but also on how we behave towards them. Indeed, we tend to offer more help^17^, imitate^18^, and offer more attention and care^19^ to people that we perceive as more attractive. This bias can have practical implication, such as getting milder court sentences^20^.

In this manuscript we explore the following questions:

1. Are people positively biased in their inferential mechanisms when judging other people’s facial beauty?
2. If such a positivity bias exists, is it specific to human faces or does it apply to other entities that people judge aesthetically, such as landscapes, flowers, and pets?
3. Is it specific to aesthetic judgements, or does it carry into other evaluative dimensions (e.g., perception of warmness)?
4. Is the bias stronger for one gender?
5. Can this bias be disrupted? And
6. What is the mechanism underlying such a positivity bias?

In our first experiment, participants were instructed to judge the attractiveness of 96 human faces while being randomly assigned to one of four conditions (each corresponding to a different manipulation of facial photographs): small photographs (Small condition); photographs with only one-third of the face visible (One-third condition); blurred photographs (Blurred condition); or photographs with complete faces (Original condition). The first three conditions share the fact that they are missing information. We then compared the attractiveness ratings of the 96 faces across the four conditions, finding that participants judged, on average, small, one-third, and blurred faces as more attractive than their original counterparts. The version depicting one-third of the faces led to the largest positivity bias and the small size version led to the smallest (but still significant) bias. Moreover, we also show that the bias spills over — albeit weakened — to warmness and knowledgeableness judgments when the positivity bias for attractiveness is strong (One-third condition). In this experiment we also measured the mood of the participants as a way to show that the differences found between conditions cannot be attributed to differences in mood.

In the second experiment we replicate the effect with two new modifications: half faces and a manipulation in which groups of pixels, accounting for a third of the total image, were randomly removed from the photographs. Also, we included perfectly symmetric faces by using mirror-reversed halves to create complete faces. We find that the positivity bias replicates for the half and the randomly incomplete versions, whereas perfectly symmetric faces were rated as less attractive than their original and half counterparts. This suggests that the positivity bias is not based on people assuming perfect symmetry.

In the third experiment we show that the bias does not replicate for incomplete photographs of dog faces, landscapes, or flowers, which might suggest that the effect is specific to human faces.

In the last four experiments we investigate the mechanism underlying the reported positivity bias. In experiment four we show that while the attractiveness judgements are sensitive to expectation, positive expectations do not lead to an increase in the bias. We also rule out expectations of similarity with the self by showing that participants do not perceive themselves as more similar to the people in the incomplete photographs (experiment five). We propose that typicality (i.e., the use of a prototypical face to fill in the missing information) can be one of the mechanisms responsible for this positivity bias (experiment six). Finally, we show that in situations where human faces are more difficult to be recognized as a face (i.e., when presented upside down) the use of a prototypical face to fill in the missing information is less likely and thus, the effect is disrupted (experiment seven).

## Results

### Positivity bias effect

**Figure 1:**
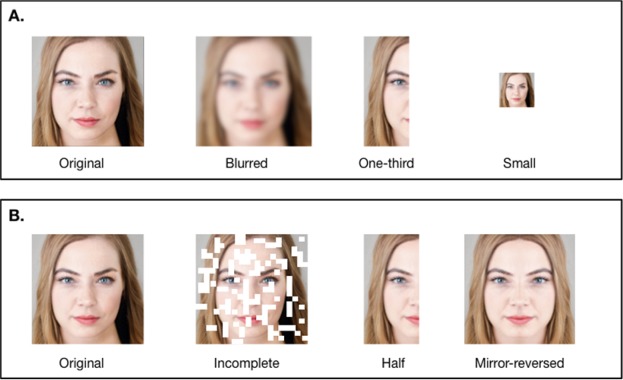
Examples of stimuli used in Experiments 1 and 2. Examples of the four manipulations used in experiment one (Original, Blurred, One-third, and Small versions-**A**) and experiment two (Original, Incomplete, Half, and Mirror–reversed-**B**). To satisfy the copyright policies of the journal, in this illustration we use an artificially generated face from the website https://www.thispersondoesnotexist.com, which uses generative adversarial networks or GANs (credited to Nvidia Corporation). However, in the experiments, we used real human faces from the website https://www.facity.com.

In the first experiment, 420 Mechanical Turk participants were presented with 96 photographs modified in one of the following ways: (1) photographs were kept in their Original format (400 × 400 pixels); (2) photographs were Blurred through the application of a 15 pixels radius Gaussian filter; (3) photographs had only One-third (the left side) of the faces visible; and (4) photographs were reduced to a Small size (50 × 50 pixels; see Fig. 1A).

All the participants were presented with the same 96 faces but they were randomly assignment to one of the four modifications. In each condition, for each photograph, participants were asked to judge how physically attractive, warm, and knowledgeable (always in this order) the people portrayed in the 96 photographs were. To give their answers, participants used a scale ranging from 1 (not at all) to 10 (very much). The response was self-paced and the mouse was used to indicate the corresponding number on the scale. At the end of the experiment participants, completed a mood scale – the Positive and Negative Affect Scale^21^.

For each of the 96 target-faces, the responses were aggregated across participants. To test if there is a positivity bias across the different modifications (Small, Blurred, and One-third) and different judgements (attractiveness, warmness, and knowledgeableness), we conducted a repeated measures ANOVA, using the average ratings of the faces as the dependent variable, and the type of judgement (attractiveness versus warmness vs. knowledgeableness) and the type of modification of the photograph (Original vs. One-third vs. Blurred vs. Small) as the two independent variables. The significant interaction found between the two independent variables, *F*(6, 90) = 55.07, *p* < 0.001, suggests that the three judgements were differently affected by the modification manipulation (see Table 1 for descriptive statistics).

**Table 1:** Descriptive statistics for all experiments.

Experiment	Modification	*M*	*SD*	*M difference*	*SE*	*95% CI*
1 - Positivity Bias effect	**Aggregated Participants: attractiveness judgements**					
	Original	4.81	1.07			
	Small	5.06	1.01	0.25***	0.06	[0.13, 0.36]
	Blurred	5.27	1.07	0.46***	0.06	[0.35, 0.57]
	One-third	5.73	1.19	0.92***	0.05	[0.81, 1.02]
	**Male Participants: attractiveness judgements**					
	Original	4.77	1.09			
	One-third	5.72	1.14	0.95***	0.05	[0.85, 1.06]
	**Female Participants: attractiveness judgements**					
	Original	4.87	1.09			
	One-third	5.74	1.24	0.87***	0.06	[0.75, 0.98]
	**Aggregated Participants: warmness judgements**					
	Original	5.19	0.83			
	Small	5.01	0.72	−0.18***	0.05	[−0.28, −0.09]
	Blurred	5.09	0.88	−0.10**	0.04	[−0.18, −0.01]
	One-third	5.58	0.88	0.38***	0.04	[0.30, 0.47]
	**Aggregated Participants: knowledgeableness judgements**					
	Original	5.89	0.54			
	Small	5.79	0.47	−0.10**	0.04	[−0.17, −0.03]
	Blurred	5.7	0.53	−0.18**	0.03	[−0.25, −0.12]
	One-third	6.3	0.53	0.41***	0.03	[0.36, 0.47]
2 - Replication	Original	48.4	11.42			
	Half	50.45	11.83	2.05***	0.45	[1.15, 2.94]
	Incomplete	51.31	10.34	2.91***	0.4	[2.11, 3.70]
	Mirror-reversed	38.28	12.66	−10.12***	0.62	[−11.34, −8.90]
3 - Specific to Human Faces	**Dog Faces**					
	Original	71.03	8.47			
	Incomplete	67	7.18	−4.02***	0.82	[−5.65, −2.40]
	**Landscapes**					
	Original	55.62	15.44			
	Incomplete	54.44	14.65	−1.18	0.82	[−2.88, 0.44]
	**Flowers**					
	Original	66.5	10.68			
	Incomplete	62.7	10.61	−3.8***	0.82	[−5.43, −2.18]
4 – Sensitivity to	**No Expectations**					
Expectations	Original	41.2	11.61			
	Incomplete	48.28	10.71	7.08***	0.45	[6.18, 7.98]
	**High Expectations**					
	Original	47.51	11.87			
	Incomplete	50.51	11.16	3***	0.39	[2.21, 3.78]
	**Low Expectations**					
	Original	43.01	12.41			
	Incomplete	44.98	9.84	1.97***	0.49	[1.00, 2.93]
5 – Ruling out Similarity	Original	33.8	4.14			
	Incomplete	34.06	2.76	0.25	0.23	[−0.20, 0.71]
6 – The role of Typicality	**Aggregated typical (experiments two and four)**					
	Original	52.95	8.86			
	Incomplete	55.17	8.47	2.22***	0.47	[1.28, 3.15]
	**Aggregated atypical (experiments two and four)**					
	Original	39.55	9.52			
	Incomplete	44.97	9.82	5.42***	0.47	[4.48, 6.35]
7 – Disrupting the positivity bias	**Upright**					
	Original	45.18	11.44			
	Incomplete	48.72	10.64	3.54***	0.4	[2.73, 4.34]
	**90-degree-rotated**					
	Original	49.63	11.48			
	Incomplete	49.94	8.53	0.31	0.48	[−0.63, 1.26]
	**Inverted**					
	Original	50.11	11.37			
	Incomplete	50.09	10.39	−0.02	0.53	[−1.06, 1.02]

Means, Standard Deviations, Means of differences, Standard Error on the Means, and Confidence Intervals on the Means, as a function of the conditions in all seven experiments. **Stands for p value = <0.05 and ***p value = <0.001.

**Figure 2:**
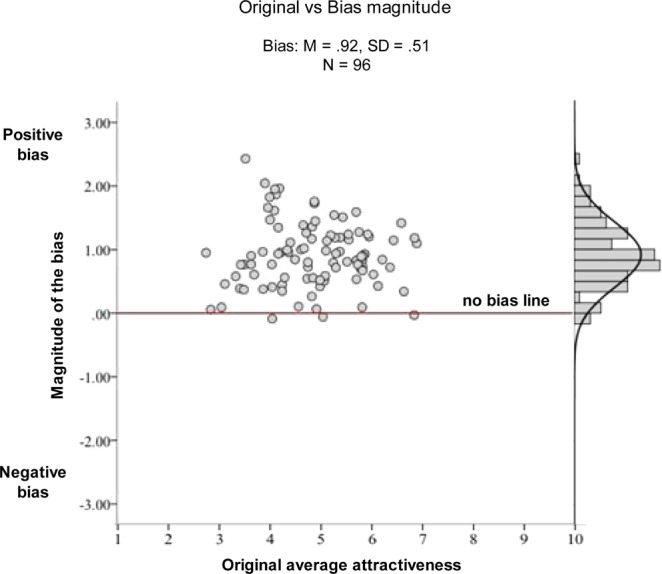
Positivity bias found in Experiment 1. The ratings for the Original faces (x axis) are plotted against the magnitude of the bias (y axis). Each dot represents one of the 96 faces.

Figure 2 illustrates the positivity bias found for the One-third condition. In this condition, incomplete faces were rated—on average—almost an entire point higher on the ten points scale than their respective original versions (*M*_*difference*_ = 0.92, *p* < 0.001). In the figure, we plotted the difference between the ratings in each of the three incomplete conditions and the ratings in the Original condition as our measure of attractiveness bias. The figure also shows that the bias is as large as two points on the scale in the strongest cases and non-existent in a handful of cases.

Participants also rated faces as less attractive in the Original condition than in the Small conditions, *M*_*difference*_ = 0.25, p < 0.001, or in the Blurred condition, *M*_*difference*_ = 0.46, p < 0.001. Among the three conditions, the One-third condition led to the largest positivity bias and the Small modification led to the smallest bias.

For warmness and knowledgeableness judgements, a negativity bias was found in the Small and Blurred conditions, since the ratings were larger for the Original faces than for the Small faces (warmness: *M*_*difference*_ = −0.18, *p* < 0.001; knowledgeableness: *M*_*differenc*e_ = −0.10, *p* = 0.008) or the Blurred faces (warmness: *M*_*difference*_ = −0.10, *p* = 0.024; knowledgeableness: *M*_*differenc*e_ = −0.18, *p* < 0.001). However, the ratings in the One-third condition were larger than in the Original condition, (warmness: *M*_*difference*_ = 0.38, *p* < 0.001; knowledgeableness: *M*_*difference*_ = 0.41, *p* < 0.001), meaning that the positivity bias found for attractiveness generalizes to warmness and knowledgeableness in this case.

Although we know, from previous studies, that men and women usually agree on attractiveness evaluations^12^, we asked whether the positivity bias is stronger for male or female participants and whether it is affected by the gender of the person being evaluated. To answer this question, we calculated the average attractiveness ratings provided by male and female participants to faces of women and men in the Original and One-third conditions. We found a small but significant interaction between the modification of the face and the gender of the participants, *F*(1,94) = 4.87, *p* = 0.03. This interaction suggests that male participants exhibit a slightly stronger positivity bias, *M*_*difference*_ = 0.95, *p* < 0.001, than female participants, *M*_*difference*_ = 0.87, *p* < 0.001. No effect of the gender of the face being evaluated was found.

We also compared the scores on the mood scale for the four conditions to assure that the differences found are not due to differences in the participants’ mood. One could argue that the effect could be a consequence of participants in the incomplete conditions enjoying more the task which could lead to more positive evaluations of the faces. Such an argument is consistent with the literature that shows hedonic states following interruptions or uncertain situations^22,23^.

Two mixed effects ANOVAs were conducted, with the modification being the independent variable and the ratings to the Positive and the Negative Affect Scales being the two dependent variables. For the Positive Scale, there was no significant effect of the type of modification, *F*(3, 413) = 0.853, *p* = 0.466, and the same is true for the Negative Scale, *F*(3, 416) = 0.691, *p* = 0.588. This result suggests that there is no reason to believe that the incompleteness of the photographs led to differences in participants’ mood.

The results of this first experiment support our hypothesis that people are positively biased when judging other people’s facial attractiveness under information shortage. Yet, this first experiment has limitations. The Blurred and the Small versions are likely to lead to objectively more attractive faces since facial imperfections, such as pimples or wrinkles, are less visible. In Experiment 2 we try to overcome this limitation by creating a new incomplete version of the photographs in which groups of pixels are eliminated at random.

291 Mechanical Turk workers took part in the second experiment. To create the material for the new incomplete condition, we divided each original photograph (400 × 400 pixels) in 400 squares of 20 by 20 pixels each and eliminated randomly a set of 150 squares from the total of 400 squares (this modification will be called Incomplete from now on). This process was repeated 100 times for each face. Two other versions were created for this experiment: Half-faces (as opposed to the One-third from Experiment 1) and Mirror-reversed symmetric faces. For the Half-face condition, as the name indicates, we cut the faces in two halves. This was done by using the equidistant point between the eyes, the central axis of the nose, and the upper lip as references. Additionally, for each face, we used these halves to create symmetric faces by combining one half face with its mirror-reversed version (see Fig. 1B for an example).

Participants were assigned to one of four conditions: Original, Half, Mirror-reversed, and Incomplete. In the Incomplete condition, for each face (and individually for each participant), an incomplete version of the face was drawn at random from the set of 100 different incomplete versions. This procedure ensures that the obtained results are not an artifact of occluding a specific facial feature in the incomplete version, because the features shown or hidden vary at random across participants. This time, participants made only attractiveness judgements and, for that, they used a scale ranging from zero (very unattractive) to 100 (very attractive).

**Figure 3:**
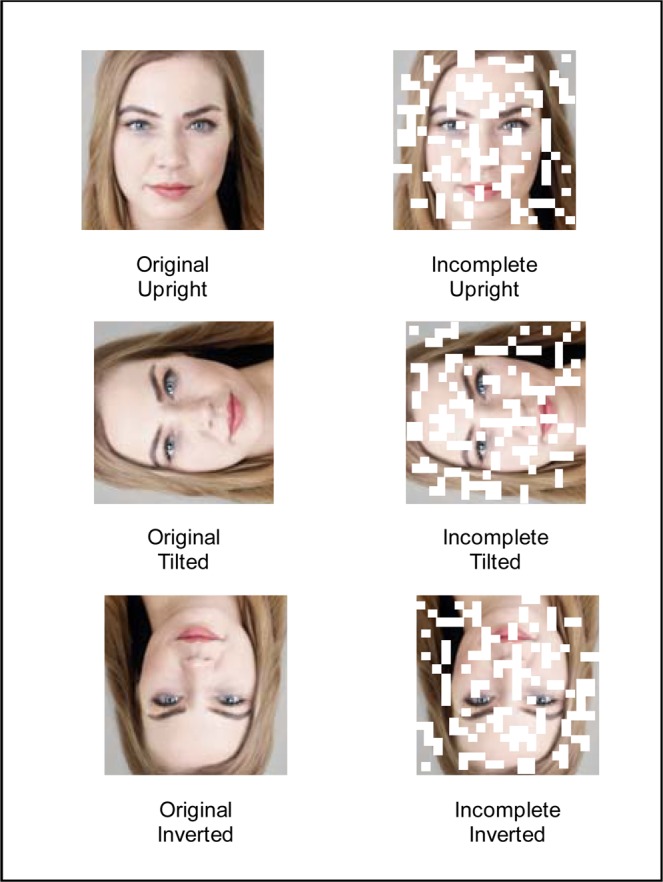
Examples of the stimuli and the manipulations in Experiment 7.

Again, we conducted a repeated measures ANOVA to test for differences across the multiple conditions (Original vs. Incomplete vs. Half vs. Mirror-reversed). We found a main effect of modification, *F*(3, 93) = 243.17, *p* < 0.001, meaning the attractiveness ratings varied significantly across conditions. The Original faces received lower ratings than Half-faces, *M*_*difference*_ = 2.05, *p* < 0.001, and Incomplete faces, *M*_*difference*_ = 2.91, *p* < 0.001, meaning the positivity bias was replicated for these new incomplete conditions. Perfectly symmetric faces, on the other hand, received ratings that were significantly lower than their Original (*M*_*difference*_ = −10.12, *p* < 0.001; see Fig. 3A) and their Half-face counterparts (*M*_*difference*_ = −12.65, *p* < 0.001). The fact that participants rated differently perfectly symmetric faces and half-faces suggests that the process taking place in the Half-face condition is probably not based on inferring perfect symmetry (inferring the missing half from the half provided; see Table 2 for means and standard deviations).

**Table 2:** Description of the sample in each experiment.

	Exp. 1	Exp. 2	Exp. 3	Exp. 4	Exp. 5	Exp. 6	Exp. 7
Sample size	417	289	205	406	223	145	413
Average age	33.4	32.02	32.04	32	32.01	31.39	32.12
SD age	7.39	6.97	6.54	7.61	6.61	6.01	7.59
Females	217	126	97	202	77	56	199
White-Americans	310	213	157	296	149	95	293
African-Americans	45	30	20	38	33	20	44
Asian-Americans	31	16	12	35	9	16	33
Hispanic-Americans	24	25	12	29	24	7	33
Native-Americans	3	0	0	1	5	5	3
Others	4	5	4	9	3	2	7
Eliminated (attention-check)	3	2	2	16	6	6	11
Compensations (in dollars)	4	2.50	2.50	2.50	1.70	1.70	2.50

### Specific to human faces

In the third experiment we used photographs of dog faces, flowers, and landscapes to test whether the positivity bias observed in Experiments 1 and 2 is also observed in these categories or whether it is specific to human faces. Dog faces are especially relevant because they are structurally similar to human faces in the sense that they have similar elements (eyes, nose, and mouth).

We had 28 photographs for each of the three categories (dogs, flowers, and landscapes) and we also generated 100 incomplete versions for each photograph through a procedure equivalent to the one used in Experiment 2. Dog faces were collected from Google using the key words: “dog faces on white background”. The landscapes and flowers were collected from McGill Calibrated Color Image Database^24^. The photos were then cropped to preserve only the area of interest (the face for the dogs and the flower for the plants). The photographs were centered and resize to 350 by 350 pixels.

207 Mechanical Turk participants were assigned to one of two conditions: Original or Incomplete photographs. For dog faces, participants were asked “how cute is the dog?”, for flowers “how beautiful is the flower?”, and for landscapes “how attractive is the scenery?”. All participants rated the dogs, the flowers, and the landscapes, in blocks. The orders of the blocks and the photographs within each block were randomized for each participant. To give their answers, participants rated the photographs on a scale from zero (not at all) to 100 (very much).

A mixed effects ANOVA revealed an interaction between the category of the stimulus and the modification, *F*(2, 81) = 3.73, *p* = 0.028, indicating that the bias was different for the three categories. For dog faces, the ratings given to the Incomplete photographs were lower than the ratings given to the Original photographs (*M*_*difference*_ = −4.02, *p* < 0.001) and a similar negativity bias was detected for flowers (*M*_*difference*_ = −3.80, *p* < 0.001). No bias was found for landscapes (*M*_*difference*_ = −1.18, *p* = 0.151). These results show that the positivity bias found for human faces does not generalize to dog faces, landscapes, and flowers. This result also agrees with past research, including Sear’s seminal paper^9^ about person-positivity bias, where the author argues that stimuli are evaluated more favorably the more they resemble individual human beings.

### Sensitivity to expectation

In the fourth experiment we measure whether the positivity bias is sensitive to the perceiver’s expectation regarding the target-faces that are being evaluated. If the positivity bias occurs due to positive expectations in the incomplete condition, by telling participants that other participants evaluated the target-faces as highly attractive should enlarge the positive expectations in incomplete photographs and increase the effect. Similarly, telling participants that the target-faces were previously evaluated by others as less attractive should decrease the use of positive expectations and thus disrupt the effect.

424 Mechanical Turk participants evaluated photographs either in the Original or the Incomplete condition (with the random elimination of pixels as described in Experiment 2). The expectation manipulation consisted of three levels: High-Expectation, No-Expectation, and Low-Expectation. In the No-Expectation condition, no information was given regarding the beauty of the target. In the other two conditions, participants were told that only faces rated as above average (or below average) by other workers would be presented to them. Participants were assigned to one of six conditions: Incomplete or Original faces, with high, low, or no expectations.

The repeated measures ANOVA suggests that the effect of expectation was significant, *F*(2, 94) = 484.01, *p* < 0.001, meaning that the ratings are overall higher in the High-Expectation condition (*M*_*High-Expectation*_ = 49.01, *SD*_*High-Expectation*_ = 11.36) than in the No-Expectation condition (*M*_*No-Expectation*_ = 44.74, *SDNo-Expectation* = 10.94), *M*_*difference*_ = 4.27, *p* < 0.001, and they are higher in the No-Expectation condition in comparison to the Low-Expectation condition, *M*_*difference*_ = 0.74, *p* < 0.001 (*M*_*Low-Expectation*_ = 44.00, *SD*_*Low-Expectation*_ = 10.94). These results suggest that participants’ judgements were sensitive to the expectation manipulation. The positivity bias was also replicated in this experiment. It was the strongest in the No-Expectation condition, *M*_*difference*_ = 7.08, *p* < 0.001, reduced in the High-Expectation condition, *M*_*difference*_ = 3.00, *p* < 0.001, and reduced even further in the Low-Expectation condition, *M*_*difference*_ = 1.97, *p* < 0.001. The differences in positivity bias across conditions were also significant (*M*_*difference between no-expectation and high-expectation*_ = 4.09, *p* < 0.001, and *M*_*difference between high-expectation and low-expectation*_ = 1.03, *p* = 0.013).

These results show that positive expectations, while increasing the overall evaluations of the faces, do not increase the bias, instead they decrease the bias. Low expectations also did not eliminate the effect, only reduced it. Hence, we conclude that expectations are not the main explaining mechanism underlying the positivity bias.

This procedure of priming expectations also reduces the ambiguity that is experienced by participants in the incomplete condition and that might have contributed to the reduction of the bias. Reducing ambiguity is expected to reduce the effect (i.e., the difference between the Incomplete and the Original faces) through a recalibration of the ratings towards the expectation induced. Our rationale is that in the condition with no-expectation, no external information is given about the attractiveness of the targets and thus, the magnitude of the bias can be freely expressed in participants’ evaluations. In other words, expectations restricted the amplitude within which the cognitive bias is operating period.

### Ruling out similarity

In the fifth experiment we test the hypothesis of whether similarity to the self could be the mechanism underlying the positivity bias. Similarity has been shown to account for positivity biases towards others in some contexts; such is the case of the research conducted by Sear^9^ and Norton *et al*.^8^. When the information about a target is ambiguous or incomplete, people erroneously perceive the targets as more similar to themselves, causing an increase in liking. If a similar mechanism is happening in the condition with incomplete faces, then we should observe higher ratings of perceived similarity in the incomplete than in the original photographs.

223 Mechanical Turk participants evaluated the 96 faces after being assigned to one of two conditions: Original or Incomplete condition. For each photograph they were instructed to indicate how similar is the person’s face to their own. To give their answers, participants rated the photographs on a scale from zero (not similar at all) to 100 (very similar).

The similarity ratings for faces in the incomplete condition were not significantly different (*M* = 34.06, *SD* = 2.75) from the ratings of the original photographs (*M* = 33.80, *SD* = 4.14), *t*(95) = 1.11, *p* = 0.271. Although, this conclusion is based on a null effect, the result suggests that the two conditions do not vary in how similar participants rate the targets to the self.

### The role of typicality

When presented with incomplete information, people infer the missing pieces based on a combination of contextual inputs and knowledge from similar past experiences. When reconstructing information regarding an acquaintance, people can fill in the blanks with memories of past interactions with that person. But, how do people fill in the missing information of a stranger that they meet for the first time? In such situations, the inference will rely on a more general visual representation. One possibility is that this representation is a typical face that people have stored in their memories as a result of their extensive exposure to human faces. If that is the case, since average/typical faces are perceived to be more attractive^25,26^, the resulting inference will reflect a positivity bias (the incomplete faces will be perceived as more attractive than the complete faces).

If typicality does play a role in the positivity bias, then the magnitude of the positivity bias (i.e., the differences in the attractiveness ratings between original and incomplete photographs) is expected to be larger for atypical faces, since they are being completed based on a more attractive typical internal representation, than for incomplete typical faces, for which the rating will be more similar to attractiveness ratings attributed to the original versions. In other words, by completing the missing information of the incomplete untypical faces based on a prototypical representation, participants are sourcing elements from a face that is known to be on average more attractive. Thus, in the sixth experiment we explore the role of typicality in the positivity bias.

145 Mechanical Turk participants were asked to rate the typicality/distinctiveness of the 96 original photographs used in the previous experiments. The photographs were paired with the question “How much does this face deviate from a typical face?” Participants provided their answer on a scale from zero (does not deviate at all) to 100 (deviates very much). Lower rating on this scale mean the face is considered more typical.

These ratings were then used to investigate the positivity bias in typical versus untypical faces, which we did by comparing the perceived attractiveness of the original versus the incomplete faces given their typicality level.

We used the median of the distinctiveness ratings to split the faces into two groups: typical and atypical. These groups were used as an independent variable together with the modification (original versus incomplete photograph) and the experiment (Experiments 2 and 4) in a mixed effects ANOVA. The dependent variable was the attractiveness ratings of the 96 target photographs. In this analysis, we used the attractiveness ratings of the original and incomplete faces from the experiments 2 and 4. These were experiments with similar design and identical modification of the photographs (from Experiment 2 only the original and the incomplete conditions were used and from Experiment 4 only the no-expectation condition was included in the analysis).

A significant effect of modification was found, *F*(1,94) = 130.869, *p* < 0.001, with higher attractiveness ratings for the incomplete (*M* = 50.07, *SD* = 10.46) than for the original photographs (*M* = 46.35, *SD* = 11.36). This result replicates the patterns found in previous experiments. A strong interaction between the modification of the faces and the typicality variable was also observed, *F*(1, 94) = 23.00, *p* < 0.001. As expected, a larger positivity bias was found for the atypical faces (*M*_*difference*_ = 5.416, *p* < 0.001) than for typical faces (*M*_*difference*_ = 2.216, *p* < 0.001). We also conducted a partial correlation between typicality and the attractiveness of the incomplete faces while controlling for the attractiveness of the original faces. A significant moderate correlation was found, *r*(93) = −0.407, *n* = 96, *p* < 0.001. These results are indicative of the role of typicality in the positivity bias effect.

### Disrupting the positivity effect

In the seventh and last experiment, we test whether the positive bias can be disrupted. There is evidence in the literature that judgments of facial attractiveness rely on holistic representations of human faces^27^. Thus, we hypothesized that the positivity bias found for attractiveness judgements of incomplete faces will also depend on holistic processing. If this is true, then, we should be able to disrupt the positivity bias by disrupting the holistic processing of faces. Inverted (up-side-down) faces have been shown to disrupt holistic processing^28,29^ (but see^30^), so we created conditions with inverted faces to test this hypothesis. Moreover, disrupting the holistic processing is known to affect other types of face processing tasks such as face recognition^31^, race categorization^32^, and emotional expression recognition^33^, among others. One possibility is that, by disrupting the holistic processing of the target-faces, participants are less successful in using the typical face to fill in the missing information, and as such, the positivity effect will not be observed anymore. In agreement with this hypothesis, judgements of distinctiveness or typicality were shown to be highly affected when the faces are inverted^34^. On the same note, Dimond and Carey^35^ proposed in 1989 that with experience, people develop fine-tuned prototypes of faces (or any other stimuli as long as a certain level of expertise is reached) that help them to encode configurational information in faces. If that is the case, then inverting the faces might disrupt the use of this prototypical spatial configuration.

422 Mechanical Turk participants took part in this experiment. The material was the same material as in the previous experiment (Original and randomly generated Incomplete versions) plus four additional versions of the 96 faces: photographs rotated 90 degrees clockwise and their corresponding Incomplete versions (100 randomly incomplete photographs for each rotated face), and 96 Inverted photographs (180 degrees rotation) and their corresponding Incomplete versions (see Fig. 3B for an example).

Participants judged the attractiveness of the faces on a scale from zero (not attractive at all) to 100 (very attractive). The two independent variables in this experiment were the modification with two levels (Original vs. Incomplete) and the orientation of the photographs with three levels (Upright vs. 90-degree-rotated vs. Inverted).

**Figure 4:**
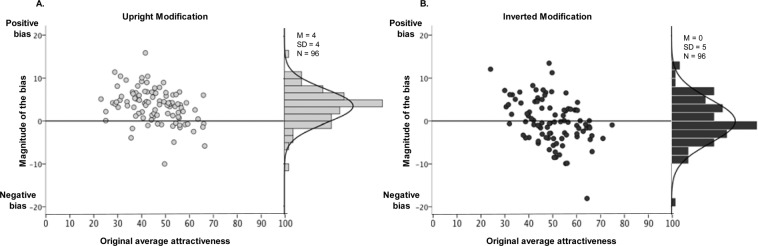
Positivity bias in Experiment 7. The positivity bias in the Upright condition (**A**) and the absence of the bias in the Inverted condition (**B**). The histograms correspond to the differences between the incomplete and the original versions in Experiment 7.

The interaction found between modification and rotation, *F*(2, 94) = 36.45, *p* = 0.028, reflects the presence of the positivity bias for the Upright photographs (*M*_*difference*_ = 3.54, *p* < 0.001), and the lack of bias for the 90-degree rotated (*M*_*difference*_ = 0.31, *p* = 0.511) and Inverted photographs (*M*_*difference*_ = −0.02, *p* = 0.968; see Fig. 4).

This experiment shows that by inverting the faces the positivity bias is disrupted, which support our hypothesis that in the inverted condition the typicality is less likely to be used to fill in the missing information.

## Discussion

We often judge others based on their physical appearance. Such judgments are driven by inferential mechanisms that help us fill in missing information. Here, we showed that (i) the inferential mechanism that we use to judge the physical appearance of human faces is positively biased, (ii) the bias is more pronounced in male participants, (iii) is specific to aesthetic judgments, but generalizes to other dimensions when the bias is strong enough, (iv) seems to be specific to human faces when compared to dog faces, landscapes, and flowers, and (v) is driven by the use of a holistic representation of what is a typical/average face. We also ruled out similarity to the self, positive expectations, and mood differences as explanatory mechanisms for the effect.

Presented with an incomplete human faces and instructed to judge their attractiveness, participants resort to what they know about faces (structure and features) and their representation of a prototypical face to generate new holistic representations. An inferential process that stems from matching the type of stimuli – i.e., human faces – with a prototype already existent in their memories. While incomplete human faces lead to an overall positive bias effect, stimuli such landscapes, pets, and flowers showed not positivity bias, which is likely due to the absence of a clear prototypical representations of these stimuli in people’s memories. Although our experiments suggest that typicality may have a role in the attractiveness positivity effect, further and more direct evidence is necessary to prove the robustness of this relationship. If typicality does play a relevant role, is also important to better understand how is this prototypical representation created and what are exactly the past experiences that shape it.

While the hypothesis that people fill in the missing pieces with positive inferences was never explicitly raised and tested, Saegusa and Watanabe stumbled on similar findings while investigating other phenomena. In their research on how information from individual facial parts contributes to the judgements of whole-face attractiveness over time, they found that attractiveness was higher for independent facial parts (e.g., eye, mouth) than for whole-faces^36^. Another study found that, on average, back-view photographs were rated as more attractive than front-view photographs^37^. The back-view condition can be seen as an extreme case of our incomplete treatments, in which the only information provided about the person is the shape of the head and the hair type, color, cut, and length. On a similar note, Miyazaki and Kawahara^38^ in an attempt to look into how the use of sanitary-masks by the Japanese women affects people’s perception of their beauty and health, found that certain types of occlusions also lead to higher perceived attractiveness, but only for originally unattractive faces judgements. Finally, Lu and collaborators^39^ manipulated the amount of information and attractiveness of cartoon characters (computer generated, gouache, and stick-figures), with the purpose of studying gender difference in attractiveness judgements. However, no significant differences were found between attractiveness judgements of the three types of cartoons. Overall, these findings support our hypothesis: when perceiving incomplete faces people fill in the missing information with positive details. Also, noteworthy, but in a domain different from that of facial perception, the work by Norton and colleagues^8^ showed that people perceive others’ personalities more favorably when they are provided with fewer personality traits as opposed to many.

Being positively biased about the attractiveness of strangers might have been a mechanism evolutionarily selected, as it might have facilitated social and reproductive events. However, the impact of this bias might only apply to impressions and interactions in first encounters. It is known that first impressions get diluted as we get to know and acquire more information about a person^7^. Thus, an interesting question for future research is the influence of the positivity bias on subsequent interactions with the target-person.

Whether the effect is unique to human faces also requires further research. More homogeneous categories than the ones we used need to be submitted to the same analysis to reach a more robust conclusion regarding the specificity of the positivity bias effect.

The contribution of face symmetry should also be studies in more detail. A meta-analysis performed by Rhodes in 2006^40^ tells us that symmetric faces are perceived as more attractive when they result from blending the original and mirror-reversed images, but they are not when they are “chimeras” (pure mirror-reversed with no blending). Pure mirror-reversed photographs lead to less attractive exemplars due to enlargement or reduction of the mid-line features^41^. In our second experiment, we used chimeras because we wanted to understand if one half of the face is used to infer the missing half, but it would be interesting to test whether using a blended symmetric face (and thus more naturally looking) would lead to a similar conclusion.

One limitation of our work is that all experiments were performed online with Mechanical Turk participants. While there is research showing that data from online experiments is comparable to data from lab-based experiments^42,43^, these conclusions need to be replicated in the laboratory and in contexts where the implications of the research might be directly relevant (e.g., social media, recruitment, fashion industry, entertainment, advertisement, and marketing).

## Methods

This research was approved by the MIT Committee of the Use of Humans as Experimental Subjects (Protocol # 1701822572). All the reported experiments were performed in accordance with the Federal regulations, 45 CFR Part 46.101(b)(2).

### Participants

The participants were all Mechanical Turk workers. They were compensated for their participations accordingly to the duration of the experiments (see Table 2) and all of them singed an informed consent form before starting. The samples sizes in all the experiments were defined a priori by using an arbitrary minimum of 70 participants per condition, and the data collection was only stopped when this number was reached for each condition of the experimental design. During the experiments, participants had to answer an attention check question, which allowed us to eliminate workers that did not pay attention to the instruction.

### Human faces stimuli

A pilot study was conducted to select the material for six of the seven experiments (1, 2, 4, 5, 6, and 7). 14 people took part in the pilot study, from which five were women. All 14 were participating in a summer school that the first author was also attending and all of them were blind to the goal of the research. The average age of this sample is 29.21 (SD = 4.46). Participants were given 200 colorful unmodified photographs (400 × 400 pixels resolution in printed format). 100 photographs depicted adult women and the other 100 depicted adult men. The photographs were downloaded from http://www.facity.com, a website that contains 4265 faces shot under similar conditions (frontal position, open eyes, natural expression – no smile, hair pulled back, none or minimum make-up, no glasses, jewelry or clothing visible, daylight, clear background, aperture 2.8 with 50 mm lens and square format). Participants were instructed to sort them in five piles: (1) very unattractive; (2) unattractive; (3) medium; (4) attractive; and (5) very attractive (ordered from very unattractive on the left to very attractive on the right on a big table). They were given 30 minutes to perform the sorting. Importantly, all participants were given only one exemplar of each face, meaning that a face could only be assigned to one of the five categories. Next, to each category we attributed an attractiveness score from to 2 to −2 (very attractive = 2, attractive = 1, medium = 0, unattractive = −1, very unattractive = −2). To obtain a single attractiveness index for each face, we calculated the weighted average of the scores, that is, the sum of the five products between the assigned value to each category and the proportion of people that attributed the face to that category:

$${A}_{i}=\mathop{\sum }\limits_{i}^{n}{a}_{i}{f}_{i}$$

where ${a}_{i}=\{-2,-1,0,1,2\}$ and ${f}_{i}=\{\frac{{n}_{-2}}{N},\frac{{n}_{-1}}{N},\frac{{n}_{0}}{N},\frac{{n}_{1}}{N},\frac{{n}_{2}}{N}\}$. A low value on this index means that the face is considered very unattractive by this group of participants and a high value means that the face is considered very attractive. From the 200 faces we selected a smaller set that would contain faces well distributed across the five categories. To do that, the faces were ordered in terms of their attractiveness index and then divided in five equal size groups (20 female and 20 male faces in each group). From these 20, we selected the 11 faces with the highest attractiveness agreement between the participants. To quantify the categorization agreement between participants, we computed the Shannon Entropy for each photograph individually:

$$S=\sum _{i}{P}_{i}lo{g}_{2}{P}_{i}$$

where *P*_*i*_ = *f*_*i*_ (see *f*_*i*_ above) and $\sum _{i}{P}_{i}=1$. A low entropy, in this context, means that people agreed with each other, and high entropy means people did not agree on the categorization of the face.

This procedure allowed us to select a final set of 106 photographs (53 female and 53 male faces) that were used in Experiments 1, 2, 4, 5, 6, and 7. Ten of these faces (five males and five females) were used in the calibrations phase described below and the remaining 96 were used in the experimental trials.

### Experimental design

In all experiments, after signing the consent form, participants answered demographic questions. Next, they completed the calibration phase, during which they evaluated the attractiveness of ten faces (in their original format) to become familiar with the range of attractiveness used in the experiment.

The Experimental Design in each experiment was as follows:

*Experiments 1 and 2*: 4 modifications (Original vs. Blurred vs. One-third vs. Small) × 3 types of judgements (attractiveness vs. knowledgeableness vs. warmness). The first variable was manipulated between-Subjects and the second within-Subject. In the second experiment, we had 4 modifications (Original vs. Incomplete vs. Half-faces vs. Mirror-reversed) manipulated between-Subjects and the judgement performed was on physical attractiveness.

*Experiment 3*: 2 modifications (Original vs. Incomplete) x 3 categories of stimuli (Dogs vs. Flowers vs. Landscapes), the judgement being aesthetic. The first variable was between-Subjects and the second was within-Subjects.

*Experiment 4*: 2 modifications (Original vs. Incomplete) x 3 types of expectations (No Expectation vs. High Expectation vs. Low Expectation), the judgement being about attractiveness. Both variables were between-Subjects.

*Experiments 5 and 6*: 2 modifications (Original vs. Incomplete) with the judgement being about attractiveness. The variable was between-Subjects.

*Experiment 7*: 2 modifications (Original vs. Incomplete) x 3 types of rotations (Upright vs. 90-degree vs. Inverted), the judgement being about attractiveness. Both variables were between-Subjects.

## Data Availability

All the data and material used will be made available upon publication.
